# Eradication of *Lomentospora prolificans* Osteomyelitis of the Wrist with Combination Antifungal Therapy, Voriconazole Bone Cement, and Surgical Debridement

**DOI:** 10.1155/2020/8271471

**Published:** 2020-09-17

**Authors:** Jeremy Lee, Mark Wilson, Nikki Casey

**Affiliations:** Orthopaedic Department, Sunshine Coast University Hospital, 6 Doherty Street, Birtinya, Queensland, Australia 4575

## Abstract

*Lomentospora prolificans* is an emerging pathogen that is difficult to treat due to its intrinsic resistance to currently available antifungal agents. Current evidence demonstrates synergy between Azoles and Terbinafine against *L. prolificans* infections, while adjunct use of antifungal agent-loaded bone cement has also shown favourable outcomes. We report a case of an immunosuppressed adult with rheumatoid arthritis who developed *L. prolificans* osteomyelitis in his right wrist following trauma and subsequent exposure to commercially available fertiliser. The infection was successfully eradicated via a combination of aggressive, staged surgical source control, antifungal therapy using voriconazole and Terbinafine, and insertion of voriconazole-loaded bone cement into the wrist and carpus. The utility of this approach supports the synergistic effects of voriconazole and Terbinafine and, more broadly, the clinical benefits of antifungal-loaded bone cement, as demonstrated in previous case reports and in vitro studies. As such, combination antifungal therapy and voriconazole-loaded bone cement should be considered the therapy of choice in cases of osteomyelitis where *L. prolificans* is proven to be the causative organism.

## 1. Introduction

*Lomentospora prolificans* is a saprophytic fungus commonly found in soils, manure, compost, and polluted waters, usually in countries with arid climates such as Australia [1–4]. Since its first documented case as a pathogen for human disease in 1984, *Lomentospora prolificans* has also been known as *Scedosporium inflatum* and *Scedosporium prolificans* [5, 6]. It is now appreciated that *L. prolificans* has a wide range of clinical manifestations that vary from localised infection in the lung, soft tissues, bones, and joints to a disseminated form that can be fatal, especially in immunocompromised populations, where mortality rates approach 80% [1, 4, 7, 8]. Fungal osteomyelitis is an example of such a pernicious manifestation, which is often preceded by trauma that disrupts the anatomic barrier to allow the pathogen to inoculate the region in question [1, 4, 7]. The standard goal of therapy involves meticulous, yet aggressive, surgical source control and antibiotic treatment, utilising the best available guidelines, along with infectious disease specialist support; the minimisation of joint and soft tissue dead space and balanced, multidisciplinary management of often significant medical comorbidities, including diabetes, heart disease, and rheumatological conditions, form an important adjunct to these broad objectives [9–11]. While *L. prolificans* has intrinsic resistance to all available antifungal agents, including the Azoles, Terbinafine, and Amphotericin B, several case reports and in vitro studies have shown favourable outcomes with combination antifungal therapy, notably voriconazole with Terbinafine [1, 8, 12–20]. Additionally, in vitro studies have also shown the potential benefits of using antifungal-loaded cement beads, which are made by the surgical team in theatre, as an adjunct to antifungal therapy, with case reports showing variable measures of success [9, 21–23]. This case report describes *L. prolificans* osteomyelitis of the wrist and carpus in an immunocompromised adult male host, successfully treated with combination antifungal therapy, staged surgical debridement, and the use of intraoperatively made voriconazole-loaded cement beads.

## 2. Case Presentation

A 68-year-old male with a background of rheumatoid arthritis (RA) presented to the Emergency Department (ED) in August 2017 with a six-week history of pain, erythema, and swelling over the dorsum of his right hand and wrist, with subjective fever and chills. The patient initially sustained small abrasions to the right dorsal hand, volar aspect of the index finger, and lateral aspect of the fifth metacarpophalangeal joint from fish spines while ocean fishing, with further injuries from a tree branch while gardening the next day. These sites became painful with necrotic foci and purulent discharge, and erythema and swelling began spreading to the rest of the hand and wrist over subsequent days. Ultrasound of the right wrist via the patient's local doctor showed features consistent with cellulitis and generalised tenosynovitis of the flexor tendons and the extensor compartment. However, multiple courses of oral antibiotics (erythromycin, cephalexin, and Flucloxacillin, in turn) did not provide any clinical improvement. The patient had previously been well, with his RA, diagnosed in 2015, well-controlled on weekly methotrexate (10 mg) and etanercept injections (50 mg) and daily prednisolone (7.5 mg) and hydrochloroquine (200 mg).

On examination, the patient had necrotic lesions over the sites of injury and extensive oedema over the right dorsum of the hand and wrist. He had negative Kanavel's signs for flexor tenosynovitis and nonirritable carpal, metacarpal, proximal, and distal interphalangeal joints. His inflammatory markers were elevated (CRP 80 mg/L, ESR 80 mm/hr, WCC 12.6 × 10^9^/L, and neutrophils 10.69 × 10^9^/L), but he was afebrile and had negative blood cultures. Furthermore, MRI of his right hand showed extensive synovitis and flexor group enhancement, but follow-up labelled white cell scan was negative for deep infection or osteomyelitis. Excisional biopsies of his necrotic lesions did not identify any organisms. The patient was given intravenous (IV) Flucloxacillin and oral doxycycline; however, there was no clinical improvement despite seven days of treatment in total since initial clinical presentation. An atypical organism as the underlying aetiology was suspected given the patient's immunocompromised state and exposure to commercially available fertiliser product, and so, the patient's RA medications were ceased, and right wrist synovectomy with biopsy was organised to identify the organism.

**Figure 1:**
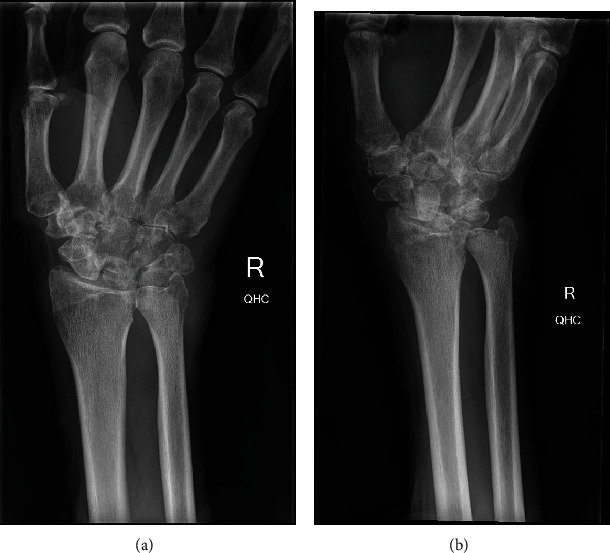
XR of the right hand post open synovectomy and biopsy: AP (a) and lateral (b) views. Erosive arthropathy at the intercarpal joints and carpometacarpal joints, with joint space narrowing within the radiocarpal articulation. Cortical erosion and reduced trabecular markings within the base of the 5^th^ metacarpal and ulnar styloid.

Open synovectomy of the right wrist with biopsy was done in January 2018, which showed extensive arthritis of the distal radioulnar joint without any evidence of organisms on microbiology, culture, and sensitivities (MCS); myriad samples were taken intraoperatively. Follow-up X-ray and MRI of the right hand in April 2018 showed changes favouring acute or chronic rheumatoid arthritis, without joint effusion or drainable collections, while the patient continued to have pain and swelling over the dorsal and increasingly over the volar wrist with associated reduced range of motion (Figure 1). Diagnosis of an acute rheumatoid arthritis “flare” was established given the recently ceased RA medications and negative microbiology results, and so, the patient was booked for a right wrist fusion with iliac crest graft for definitive management of his pain.

However, when the patient presented for the procedure in November 2018, a fluctuant collection under the previous synovectomy scar was noted. Upon incision, purulent material was discharged from the joint space, with extensive synovitis and destroyed distal radioulnar joint noted in the operation report. Surgical debridement and irrigation were performed instead, with multiple tissue and synovium samples sent for microbiology, culture, and sensitivities (MCS).

Microbiology was positive for *Enterobacter cloacae* and *Lomentospora prolificans*. The identified *E. cloacae* was resistant to co-trimoxazole and sensitive to gentamicin, ciprofloxacin, and Meropenem. Minimum inhibitory concentration (MIC) for *L. prolificans* was performed via the broth dilution method at a specialized mycology unit interstate, which showed MIC of >8 mg/L to Amphotericin, >256 mg/L to fluconazole, >16 mg/L to itraconazole, and >8 mg/L to voriconazole. During this clinical presentation, the patient's CRP was 14 mg/L and WCC 8.0 × 10^9^/L. Following surgery, the patient was started on IV Meropenem (1 g TDS) with oral voriconazole (250 mg BD) and Terbinafine (250 mg BD), as per the recommendations from the infectious disease (ID) team. The trough voriconazole level was monitored weekly to ensure that the given dose was within the therapeutic range for the patient.

**Figure 2:**
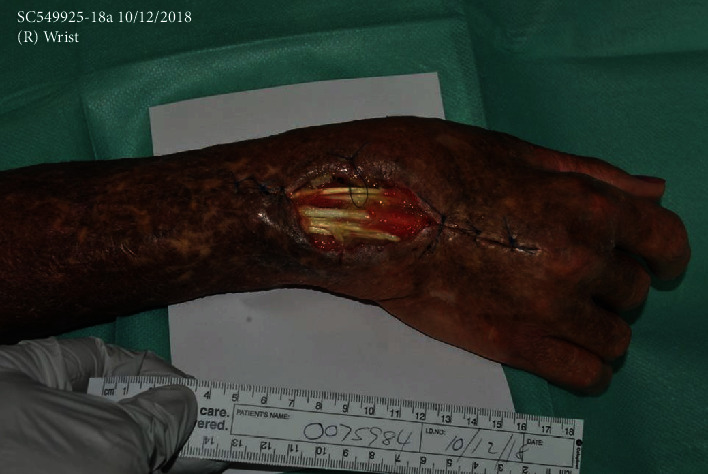
Clinical photograph of the right hand in between serial surgical debridement and washout. Note the extensive soft tissue damage with exposed extensor tendons.

Surgical debridement and irrigation were repeated every three to five days to regain source control. There was extensive damage from the disease process requiring extensor tendon synovectomy and eventual proximal and distal row carpectomy. The skin overlying the wrist had necrotic, sloughed edges that required progressive debridement, resulting in a large dorsal wrist wound with exposed, residual extensor tendons (Figure 2). This was deemed a nongraftable surface by the plastic surgical team until the infection was resolved, despite the prolonged time course. Due to the extent of bony and soft tissue damage and the possibility of upper limb amputation at a suitable level, should the infection not be controlled locally, advice was sought from local orthopaedic colleagues regarding the use of antifungal-impregnated cement beads as an adjunctive therapy. Voriconazole was recommended as the agent of choice to be incorporated into the drug-eluting cement beads fashioned intraoperatively.

**Figure 3:**
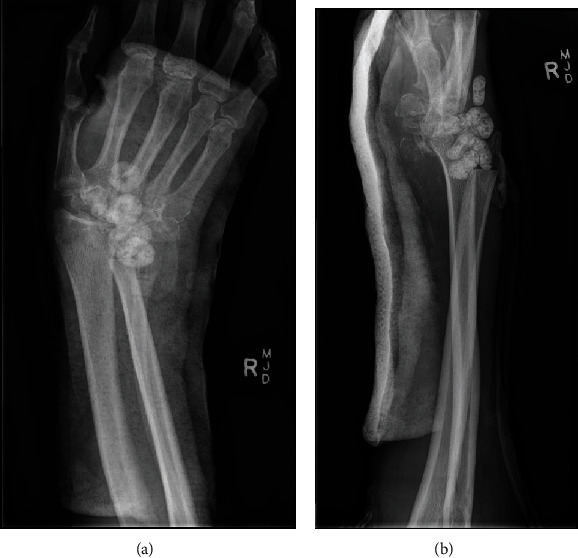
XR of the right hand with voriconazole-impregnated Palacos beads in situ: AP (a) and lateral (b) views. Note the evidence of proximal carpectomy and distal ulnar excision.

**Figure 4:**
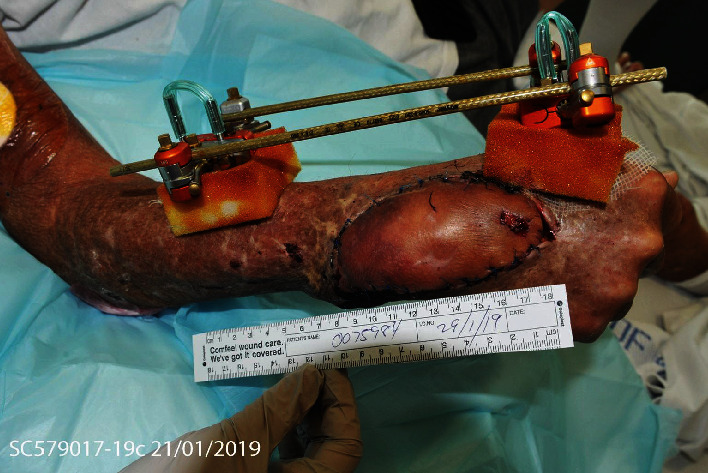
Clinical photograph of the right hand with external fixation devices in situ.

**Figure 5:**
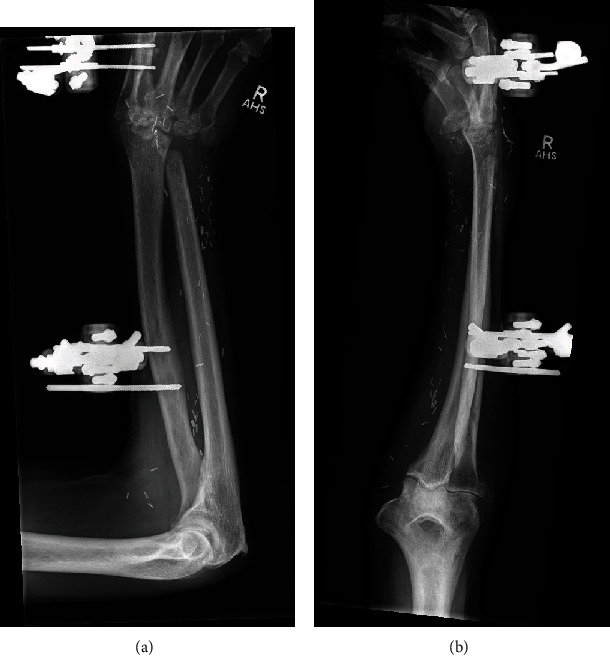
XR of the right hand with the external fixation device in situ: AP (a) and lateral (b) views. Proximal screws in the midright radius, distal screws in the second metacarpal.

Seven Palacos (nonbiodegradable) cement beads impregnated with 600 mg of voriconazole were inserted in December 2018 during proximal row carpectomy, washout, and debridement (Figure 3). A total of nine sessions of joint irrigation and debridement were performed from November 2018 to January 2019. The Palacos beads were in situ for a total of three weeks before they were removed at one of the above sessions. Samples taken since the insertion of voriconazole beads were consistently negative for *L. prolificans* on microbiology, and the right wrist showed clinical improvement, eventually to complete resolution of pain with no ongoing signs of infection. External fixation was applied to the now flail right wrist in January 2019 to achieve skeletal stability, and the patient was discharged on oral voriconazole and Terbinafine (Figures 4 and 5).

**Figure 6:**
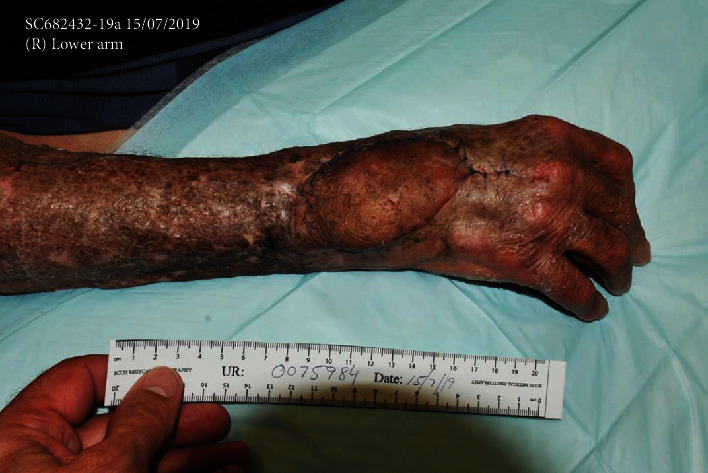
Clinical photograph of the right hand, post wrist fusion.

The patient remained well in subsequent outpatient reviews and was eventually deemed fit for right wrist fusion with iliac crest bone graft and internal fixation, which was completed in July 2019. Voriconazole-impregnated Stimulan (biodegradable) was used throughout the deep dead space during the procedure. The plastics team performed a myocutaneous flap to close the dorsal wrist wound. At four months post right wrist fusion, the patient remained clinically well and pain-free and had regained some function in relation to the use of the right hand (Figure 6). Follow-up imaging showed a stable right wrist post fusion with no evidence of collection or hardware failure. The patient is currently on voriconazole (200 mg BD) and Terbinafine (250 mg BD), with regular trough voriconazole levels monitored by the ID team; he is to complete at least 12 months of oral voriconazole in total, before cessation of treatment. It is not believed that the patient will need life-long suppressive treatment with either antibiotics or antifungal agents at this stage.

## 3. Discussion

*Lomentospora prolificans* was isolated in 1984 when it was identified as the cause of osteomyelitis in a bone biopsy of a six-year-old's foot [5]. Despite a relatively short history, it has become an emerging pathogen of human disease, with an increasing number of cases over the past several decades in both immunocompetent and immunocompromised populations, largely due to its intrinsic resistance to currently available antifungal agents [4, 8, 12, 15].

A systematic review of 162 cases of *L. prolificans* by Rodriguez-Tudela et al. [7] showed that the pathogen commonly affects the lungs, bones, joints, and soft tissues. Disseminated infection was the most common at forty-four percent, with a high mortality rate of eighty-seven percent. Most of these patients had concurrent haematological malignancies and neutropenia, which is identified as a risk factor, demonstrating *L. prolificans*' opportunistic tendency to exploit the immunocompromised host [4, 7, 24, 25]. In contrast, around 10 percent of the patients presented with osteomyelitis and/or septic arthritis, with trauma as a common preceding factor in almost all cases [7]. This was evident in this case report patient's history of abrasions to the hand, which would have disrupted the anatomic barrier and allowed the fungus in the compost to enter. While the patient was immunosuppressed, his infection fortunately did not progress to a disseminated form, which is near-universally fatal, especially in immunocompromised hosts; this may be due to the absence of risk factors such as neutropenia and fever, which represents a much more sustained systemic inflammatory response and confers a poorer prognosis [7].

Osteomyelitis is a complex condition that requires a multidisciplinary approach between orthopaedics, infectious diseases, and plastics, as outlined in this case report [10, 11, 26]. The goal of therapy is to eradicate the infection and avoid soft tissue and functional loss, via a combination of surgical source control, antibiotic delivery, minimising joint and soft tissue dead space, and managing comorbidities [9–11, 21]. However, in vitro studies on antifungal susceptibility of *L. prolificans* consistently show high minimum inhibitory concentrations (MIC) across all antifungal agents, including Azoles, Terbinafine, and Amphotericin B [1, 12, 15]. Joint guidelines by the European Society of Clinical Microbiology and Infectious Diseases (ESCMID) and European Confederation of Medical Mycology (ECMM) recommend extensive surgical debridement with either voriconazole as a monotherapy or in combination with Terbinafine, but its quality of evidence is grade III, which is based on the opinions of respected authorities, clinical experience, and case studies only; no level 1 evidence in the form of randomised controlled clinical trials or meta-analyses is available, to our knowledge, to help inform clinicians in this important area [2]. Nonetheless, several in vitro studies have demonstrated synergistic effect of using Azoles and Terbinafine together. While *L. prolificans* against either Azoles or Terbinafine as monotherapy displays high MIC, a combination of the two agents has been shown to reduce the MIC significantly to clinically achievable concentrations [1, 13–15]. This is believed to be due to the Azoles and Terbinafine affecting different steps of fungal ergosterol biosynthesis [1]. In practice, success with the use of combination antifungal therapy has also been described in several case reports, supporting its clinical value in treating infections driven by *L. prolificans* [14, 16–20].

Bone cement as a vehicle for local delivery of antifungal agents is an additional method of overcoming the pan-resistance posed by *L. prolificans*. The technique of incorporating antibiotics into polymethylmethacrylate (PMMA) cement was first developed in 1970 by Buchholz and Engelbrecht [27] and has since been used in open fractures, osteomyelitis, and index and infected arthroplasty procedures [21, 28]. The advantage of this modality is its ability to deliver antibiotics locally, at a concentration greater than the MIC required to treat the infection at its source, which may not be achievable via the parenteral route [9, 29]. While there is the risk of local cytotoxicity and other adverse patient effects, the use of drug-eluting cement beads has minimal risk of systemic effects and does not depend on the vascularity of the target tissue [28]. Indeed, PMMA is an example of a nonbiodegradable cement, whereas Stimulan (calcium sulfate) is an example of a biodegradable counterpart; both have their described advantages and disadvantages [9, 21]. Furthermore, an in vitro study by Grimsrud et al. [21] demonstrated that both PMMA and Stimulan initially eluted a high concentration of voriconazole at a similar rate, which quickly decreased over 48 hours to a relatively consistent rate for the following two weeks. However, it is important to the cognisant that the baseline elution rate by 200 mg of voriconazole was close to the MIC of *L. prolificans*. A further in vitro study by Miller et al. [22] proved that the elution rate was dose-dependent by comparing the rate between 300 mg and 600 mg of voriconazole over thirty days. These studies suggest that voriconazole-loaded bone cement locally delivers concentrations that likely exceed the MIC of *L. prolificans* at a consistent rate, over a prolonged period, augmenting its clinical efficacy in the treatment of aggressive fungal infections.

This is the second Australian case to describe the use of voriconazole-loaded bone cement to treat *L. prolificans*-associated osteomyelitis. To our knowledge, the only other reported Australian case was by Daniele et al. [23], who used voriconazole-loaded cement and spacer to treat *L. prolificans*-driven hip osteomyelitis and septic arthritis. Their outcome was similar, achieving infection eradication and restoring function and pain relief to the patient.

## 4. Conclusion

*Lomentospora prolificans* is an opportunistic fungus capable of causing fatal infections, especially in immunocompromised and other susceptible hosts, and treatment is often complicated due to its intrinsic resistance to currently available antifungal agents. Nevertheless, current evidence suggests that combined use of voriconazole and Terbinafine works synergistically to overcome this resistance and control the infection. In addition, adjunct use of voriconazole-loaded bone cement is also shown to be a highly effective and viable mode of treatment to control the infection at the source. This case illustrates the successful outcome of these treatments, supporting their use in clinical practice, especially when combined with judicious, yet aggressive, local source control in otherwise poor surgical hosts.
